# A Retrospective Study of Non-Communicable Diseases amongst Blue-Collar Migrant Workers in Qatar

**DOI:** 10.3390/ijerph19042266

**Published:** 2022-02-17

**Authors:** Fatima Al-Hatimy, Abdulaziz Farooq, Mohamad Al Abiad, Shilpi Yerramsetti, Maryam Ali Al-Nesf, Chidambaram Manickam, Mohammed H. Al-Thani, Al-Hareth Al-Khater, Waseem Samsam, Vidya Mohamed-Ali, Mohammed Al-Maadheed

**Affiliations:** 1 Anti-Doping Laboratory Qatar, Sports City Road, Doha P. O. Box 27775, Qatar; fatima.al-hatimy.18@ucl.ac.uk (F.A.-H.); mohammed.farooq@aspetar.com (A.F.); cmanickam@adlqatar.qa (C.M.); wsamsam@adlqatar.qa (W.S.); v.mohamed-ali@ucl.ac.uk (V.M.-A.); 2 Center of Metabolism and Inflammation, Division of Medicine, Royal Free Campus, University College London, Rowland Hill Road, London NW3 2PF, UK; mariamali@hamad.qa; 3 Aspetar, Orthopaedic and Sports Medicine Hospital, FIFA Medical Centre of Excellence, Doha P. O. Box 29222, Qatar; 4 Qatar Red Crescent Society, Doha P. O. Box 5449, Qatar; mohamadalabyad@yahoo.com (M.A.A.); shilpi.yerramsetti@qrcs.org.qa (S.Y.); aalkhater1@hamad.qa (A.-H.A.-K.); 5 Hamad Medical Corporation, Doha P. O. Box 3050, Qatar; 6 Ministry of Public Health, Doha P. O. Box 42, Qatar; malthani@moph.gov.qa

**Keywords:** non-communicable diseases, diabetes, hypertension, dyslipidaemia, blue-collar workers, low-income community

## Abstract

*Background:* South Asian workers have a greater predisposition to non-communicable diseases (NCDs) that is exacerbated by migration and length of residence in host countries. *Aims:* To examine the association between length of residence in Qatar with diagnosis of NCDs in male blue-collar workers. *Methods:* A retrospective investigation of the electronic health records (EHRs) of 119,581 clinical visits by 58,342 patients was conducted. Data included age, nationality and confirmed ICD-10 diagnosis. Based on duration of residence, the population was divided into groups: ≤6 months, 6–12 months, 1–≤2 years, 2–≤5 years, 5–≤6 years, >6 years. It was assumed that the group that had been resident in Qatar for ≤6 months represented diseases that had been acquired in their countries of origin. *Results:* South Asian (90%) patients presented with NCDs at a younger (mean ± SD age of 34.8 ± 9.0 years) age. Diabetes and hypertension were higher in those who had just arrived (<6 months’ group), compared to the other durations of residence groups. Conversely, acute respiratory infections, as well as dermatitis and eczema, all increased, perhaps a consequence of shared living/working facilities. Only patients with diabetes and hypertension visited the clinic multiple times, and the cost of medication for these NCDs was affordable, relative to earnings. *Discussion/Conclusions:* Blue-collar workers were predominantly South Asian, from lower socioeconomic classes, with early onset chronic NCDs. Notably, residence in Qatar gave them better access to affordable, significantly subsidized healthcare, leading to effective management of these chronic conditions.

## 1. Introduction

Non-communicable diseases (NCDs) contribute to more than 70% of all-cause mortality globally, with potentially significant socioeconomic consequences [1]. Eighty percent of all premature NCD deaths are accounted for by cardiovascular diseases (17.9 million deaths pa), diabetes (1.6 million deaths pa), respiratory diseases (3.9 million deaths pa), and cancers (9.0 million deaths pa) [1]. The greatest burden is imposed disproportionately on low- and middle-income communities, especially blue-collar workers, both males and females. Moreover, greater numbers of NCD-related deaths occur at a younger age (below 60 years) in low-income groups (30% of deaths), as opposed to those with higher-incomes (13% of deaths) [2]. 

Overall, evidence from several parts of the world support these findings [3,4,5,6,7,8,9,10,11,12,13]. Spanish female blue-collar workers had a higher incidence of metabolic syndrome compared to white-collar personnel [3]. In China, social determinants that contribute to NCDs, such as poor education, smoking, increased alcohol consumption and air pollution, are all more prevalent amongst blue-collar workers. In addition, lower education levels and urban residency are strongly associated with an increased risk of diabetes [4]. Tobacco use, hypertension and physical inactivity are significantly more prevalent in lower education groups in India [5]. Cardiovascular mortality rates have decreased among educated people compared to those without formal education in Vietnam [6]. Poverty is also associated with harmful alcohol and tobacco use in Nepal and India [7,9,10]. In Brazil, obesity was found to be higher among less educated women [11]. Higher mortality from NCDs was found among the urban poor, and a greater risk of exposure to a number of its risk factors, including passive smoke, excessive alcohol use and indoor air pollution has been identified in South Africa [8]. Those from low–middle income may also begin life with increased vulnerability to NCDs, compounded by being exposed to additional risks throughout life. Undernutrition during the embryonic period and low birth weight, particularly prevalent among low-income populations, increases the subsequent risk of cardiovascular disease and diabetes [12]. Moreover, childhood poor socioeconomic status is associated with type II diabetes and obesity in later life [13].

Immigrant populations are disproportionally affected by NCDs, probably due to pre-existing risk factors, as well as exposure to new environmental and occupational stressors. Moreover, evidence points to an association with the duration of residence in a foreign country aggravating these risks. Diabetes, for instance, is more prevalent amongst immigrants in Australia [14] and the Netherlands [15], compared to the Europid Australian and Dutch populations, respectively. Higher mortality rates due to ischaemic heart disease have been reported in immigrants, compared to the long-term residents of Canada [16]. A higher incidence of heart disease was reported in Mexican migrants returning from the USA, compared to non-migrants in Mexico [17]. 

An area of intense current public scrutiny relates to low- to middle-income blue-collar workers that are residents in the Gulf Cooperation Countries (GCC) of the Middle East. Workers, mainly males, from countries of the Indian subcontinent are particularly attracted by the significant employment opportunities offered by the large infrastructure building projects in the GCC [18]. The prevalence of NCDs is higher in these workers. However, these are often comparable to that seen in the host GCC population, possibly due to some overlap in dietary practices, sedentary lifestyle or epigenetic factors [19,20,21]. Despite this, a longer length of residence (>10 years) has been associated with a more than two-fold increase in the prevalence of diabetes among female migrant workers in the United Arab Emirates (UAE) [18], suggesting that both migration and duration of residence in the foreign country may equally impact health outcomes. 

The current pre-employment medical examination in the GCC countries focuses on communicable diseases, such as TB and HIV, and also on those medically unfit for particular occupations, such as thalassemia and jobs associated with lead exposure [22]. However, no screening programme currently exists aimed at diagnosing NCDs or their risk factors prior to employment. While this may not be of concern in a healthy migrant population with no underlying predispositions, it is of note for those with a propensity to develop chronic diseases at a younger age [23].

Therefore, this study examined the association between the length of residence in Qatar and diagnosis of NCDs in male blue-collar workers, using a descriptive, retrospective model, to highlight pre-existing risk and the healthcare provision aimed at this vulnerable sector of the population, as well as the financial burden associated with NCDs.

## 2. Materials and Methods

### 2.1. Study Design and Settings

There are six clinics located in Doha, Qatar, within 30 min of blue-collar workers’ residences or workplaces, established to meet workers’ primary/intermediate health care needs, set up by the Qatar Red Crescent Society (QRCS), and sponsored by the Ministry of Public Health (MoPH). The clinics offer primary care specialty clinics, with internal medicine, cardiology, endocrinology, respiratory medicine, orthopaedics, nutrition, ENT, dental care, minor injuries clinics, and support services, such as radiology, laboratory and pharmacy. In addition, free access is provided to the Hamad Medical Corporation (HMC)—the main tertiary care centre—in an emergency or when further care is needed. 

Using EHRs, a retrospective study was conducted on a cohort of migrant, male blue-collar workers, resident in Qatar for a period spanning 10 years. Data focused on patients’ demographic characteristics, mainly age and nationality, and a confirmed diagnosis of NCDs based on ICD-10 [20], was collected from the EHRs of the largest of the (QRCS) health clinics (Mesaimer), based in the Industrial Area Zone, Doha, Qatar, from 1 January 2017 to 30 May 2018. 

### 2.2. Participants

Participants were all males, aged 18–65 years. Migrant workers were defined by place of birth (i.e., foreign-born), nationality (i.e., foreign citizens), and length of residence in Qatar. In this study, the foreign-born definition was based on nationality, and recent migrants were those who lived in Qatar for two years or less. The blue-collar migrant workers were defined as foreign nationals who moved to Qatar for the sole purpose of employment (Ministry of Interior, State of Qatar), on low- to middle-incomes and in occupations defined by the Ministry of Administrative Development, Labour and Social Affairs, State of Qatar. It was estimated that during the study period there were approximately 1.2 million workers in Qatar, the majority of whom were male and mainly working on construction sites. 

The duration of residence in Qatar was computed from the national patient identification number, and the matched records from the Ministry of Interior, Immigration Department, to determine the exact date of first entry to Qatar. Other supplementary data related to the study population’s estimated income was retrieved from reports and records from government contracts and information from contractors.

### 2.3. Statistical Analysis

Descriptive statistics were presented as mean and standard deviation (SD) for continuous variables and median and interquartile range (IQR) for skewed data. Categorical variables were reported as frequency and percentage. Based on the duration of residence in Qatar, the cohort was divided into six groups: ≤6 months; 6–12 months; 1–<2 years; 2–<4 years; 4–<6 years; and ≥6 years. It was assumed that the group that had been resident in Qatar for less than six months represented diseases that might have been acquired in their countries of origin. One-way analysis of variance (ANOVA) was used to compare the age and duration of residence according to ICD-10-main groups. A post-hoc analysis with Bonferroni correction was used for further comparisons in the event of significance. A chi-square test for independence was performed to compare the prevalence of ICD-10 main groups and ICD-10 subgroups according to the duration of the residence groups. Column proportions were compared for differences, and p-values were computed after adjusting for Bonferroni correction for multiple comparisons. Data was coded and analyzed using the Statistical Package for Social Sciences (SPSS, Chicago, IL, USA) for Windows Version 21. A *p* < 0.05 was considered statistically significant.

## 3. Results

Foreign workers account for almost 80% of the population, and are significantly skewed to males, as per Qatar’s census data of 2018 (Figure S1). The existing database consisted of 650,000 recorded clinic visits. Records of patients with non-availability of valid identification, date of entry into Qatar, or clinical diagnosis as per ICD-10 were excluded. Patients aged ≤18 years (n = 55) and ˃65 years (n = 246) were also removed. A final database of 181,359 recorded clinic visits was generated for the duration of the 18-month study period, in whom all variables were available and accurate, consisting of 58,270 unique patients. The study was approved by a Ministry of Public Health registered Institutional Review Board (Study Number Е2018000286). 

### 3.1. Employment

All patients were male, with 24% laborers, 8.8% drivers, 5.1% masons, 4.3% carpenters and 4.1 % general building workers, making up nearly half of the study population (46.6%). An additional 25% were directly related to the construction industry.

### 3.2. Ethnicity

Patients registered at the clinic were predominantly from the Indian sub-continent (90%), mainly India (29.5%), Nepal (28.6%) and Bangladesh (21.9%), and with 83% of the population aged 25–55 years. The remaining 10% of the patients were from Egypt, Syria, the Philippines or the African continent. Specifically, those from West and Central Asia n = 1960, North Africa n = 2149, sub-Saharan Africa n = 1819, Europe n = 7, East Asia n = 3, North Asia n = 3, and Oceania n = 1 (Table 1).

### 3.3. Body Mass Index (BMI)

In a subsample of 1013 patients, mean ± SD age 36.5 ± 9.1 years, body fat mass, an independent risk factor for chronic metabolic diseases, and BMI were assessed (mean ± SD BMI 25.3 ± 3.7 kg.m^−2^ and body fat was 21.5 ± 5.4%). BMI correlated positively with age (r = 0.27, *p* < 0.001). Patients from Egypt, on average, had higher BMI compared to patients from Bangladesh (*p* < 0.001), India (*p* = 0.001), Nepal (*p* = 0.001) and Sri Lanka (*p* = 0.02). Bangladeshi patients had lower BMI than patients from Pakistan (*p* = 0.005) and the Philippines (*p* = 0.004). 

### 3.4. Duration of Residence

The majority (84.5%) had been resident in Qatar for three or more years, with over 96% having been there for more than 12 months. Overall, the median (IQR) period of residence in Qatar was 4.1 (2.3–8.1) years, and the mean (SD) age was 37.2 (9.6) years. Younger workers (˂35 years old) were more likely to have resided in Qatar for a shorter duration, ˂4 years for those below 25 years old, and ˂6 years for those below 35 years old. Moreover, a proportionally lower number of workers were over the age of 55 years (Table 1).

### 3.5. Diagnosis by ICD-10 and Association with Duration of Residence

The cohort was categorized based on the diagnosis of disease of ICD-10 chapters for all visits throughout the study period. The top five reasons for the clinic visits were endocrine, respiratory, musculoskeletal, digestive or circulatory diseases. 

Endocrine (E00–E89) and circulatory (I00–I99) diseases were higher in those who had just arrived (<6 months’ group), compared to other groups, remaining elevated for a period of up to 6 years. Thereafter (>6 years of residence), both these diseases increased to levels comparable to or higher than in those who had just arrived. These diseases were also more prevalent in this group (by ≈2-fold) than the other duration of residence groups. 

**Figure 1:**
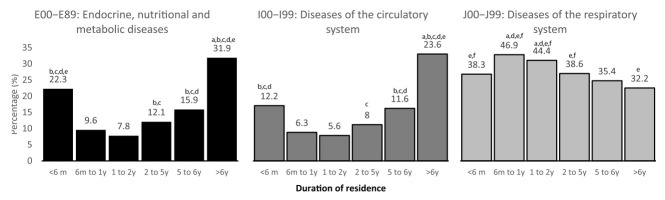
Proportion of visits based on the diagnosis of disease according to duration of residence. a: significantly higher than <6 months; b: significantly higher than 6 months to 1 year; c: significantly higher than 1 to 2 years; d: significantly higher than 2 to 5 years; e: significantly higher/lower than 5 to 6 years; f: significantly higher than >6 years.

Conversely, diseases of the respiratory system (J00–J99) were low at arrival and increased shortly thereafter for a period of up to two years. Those who had been resident in Qatar for 5 or more years had comparable or lower levels of these respiratory diseases than those who had been there for <6 months (Figure 1). 

Patients who presented with injuries, poisoning and external causes were more likely to be younger, with a mean ± SD age of 34.8 ± 9.0 years, compared to those with circulatory diseases, who were 9–12 years older than the rest of the population, closely followed by those with the endocrine diseases. The patients with circulatory diseases were the oldest group, being approximately a year older than even those with endocrine disorders (all *p* < 0.001, Table 2). They also had the highest BMI, reflecting the direct correlation between age and BMI. Furthermore, only those with endocrine and circulatory diseases were likely to have visited the clinics multiple times during any given year for treatment of chronic conditions (repeat prescriptions) and monitoring (Table 2). 

**Figure 2:**
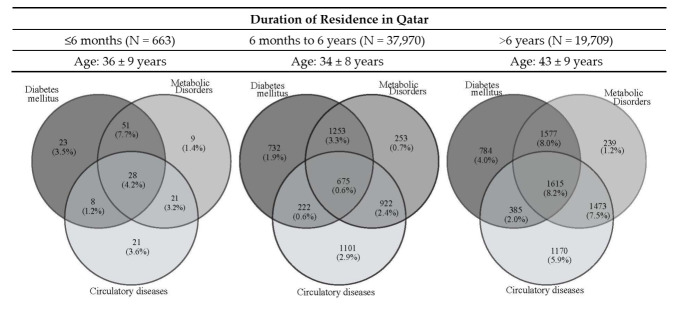
Changes in the main comorbidities with duration of residence.

Further analysis of the ICD-10 sub-groups and duration of residence with age showed that diabetes and metabolic disorders, as well as hypertension, were high on arrival (˂6 months), but lower in those who had lived in Qatar for ˃6 months up to 6 years, despite being matched for age. These diseases increased in the group that had lived in Qatar longest (˃6 years), which may be explained, at least in part, by them being significantly older and more obese (Figure 2).

Looking at other categories, respiratory and musculoskeletal complaints increased by 10% and 8–10%, respectively, in the first 6 months–1 year of being in the country. Acute upper respiratory infections and other lower respiratory infections increased with longer residence in Qatar. Similarly, dermatitis and eczema also increased, as did injuries related to the musculoskeletal system, specifically those classified as ‘other dorsopathies’ (Table 3). These conditions may be a consequence of exposure to environmental pollutants, or a lack of occupation-related training and safety. 

Patients diagnosed with injury, poisoning, or other consequences of external causes (S00-T8) seemed less likely to be affected by duration of residence; however, those that had been in the country for >6 years seemed less likely to visit clinics (Table 3). Other pathologies were relatively minor and did not vary significantly with the duration of residence in Qatar (Table S1). 

During the study period, HMC emergency records showed 7.3% of this population were admitted, of whom 5.2% were immediately discharged following a tertiary care consultation. The remaining 2.1% (n = 1224) had no discharge records and not followed up further. 

### 3.6. Income and Medical Costs

The mean (range) monthly salary of the workers was 1150 (700–1700) QAR monthly or 37.81 (23.01–55.89) QAR daily. An additional remuneration of 200 QAR was included towards monthly food and transportation costs. The clinic visits were free of charge to the patient and are borne by the government of Qatar (Table 4). The estimated cost of treating NCDs to both the individual patient and the Qatari government was calculated. The financial burden was 1.67, 1.03, 0.57, and 0.07 days’ income to the workers on the minimum wage and 0.69, 0.42, 0.24, and 0.03 days’ income to those on the maximum wage, for all NCD treatments, diabetes, hypertension, and dyslipidaemia, respectively. For those on the average monthly earnings, the cost of medication for all three NCDs to a patient was 38.34 QAR, or just over 1 days’ pay (1.01 days’ pay), while diabetes, hypertension, and dyslipidaemia were 0.62, 0.35 and 0.04 of a days’ income, independently (Table 4).

The estimated cost of treatment to the government was QAR 84.14 for an internal medicine specialist, QAR 29 for a nurse, QAR 49.40 for laboratory tests, QAR 118 for medication for diabetes, QAR 66 medication for blood pressure checks, QAR 7.70 for medication for lipids, QAR 8.70 for miscellaneous consumable items, and QAR 1.80 for ECG. The overall total cost paid by the government was QAR 524.04. The patient contributed only towards medications, amounting to QAR 38.4 (7% of total costs).

## 4. Discussion

The Middle East is the major destination for migrants from South Asia and the North African region. The GCC, in particular, is known to employ the largest number of temporary workers globally. Qatar has witnessed an increasing demand for labor, estimated to be around 79% of the population, driven by infrastructure reconstruction and preparations for the 2022 FIFA World Cup. 

This study, for the first time, demonstrated the association between NCDs and length of residence of male blue-collar workers in Qatar. It was assumed that the group that had been resident in Qatar for less than six months represented diseases that might have been acquired in their countries of origin. The diagnosis of diabetes, metabolic disorders and hypertension displayed a U-shaped relationship with duration of residence in Qatar. They were significantly higher in those who had just arrived (<6 months’ group), compared to those having lived in Qatar for 5–6 years, indicating better healthcare provision focused on these diseases. However, this improvement was lost in those having lived in the country for greater than six years. This may be, at least in part, a consequence of obesity and sedentary lifestyle, which worsens with the length of residence. 

In this study, out of 58,270 patients, 6.2% were diagnosed with endocrine, nutritional and metabolic diseases (ICD-10 E00–E80), and 8.5% with circulatory (ICD-10 I00–I99) diseases. Moreover, these were the main complications that required multiple clinic visits. These results were comparable to reports from the workers’ countries of origin. A study of morbidity patterns of outpatients reported for a city in India, showed that endocrine, nutritional and metabolic diseases accounted for 8.1% of the total patients visiting an urban healthcare training centre. Moreover, patients with circulatory diseases accounted for 27.5% [21], which was higher than that reported in this study. Possible differences could be that patients in our study were relatively younger, and only included men.

Evidence suggests that the burden of NCD is escalating in the Indian subcontinent, being diagnosed at an earlier age of ≤45 years, compared to 55 years as reported in other developed countries [22]. Additionally, there are delayed or undiagnosed cases due to lack of access to healthcare and affordability [23]. This study highlights that the average age of the migrants with endocrine and metabolic diseases resembles those of their country of origin. However, the number of clinic visits and the chances of having better medical care per patient is significantly higher. The prevalence rates of upper respiratory disorders in India increased by 29.2% for COPD and 8.6% for asthma from 1990 to 2016 [24]. The age-standardized diabetes prevalence increased by 29.7%, whereas mortality associated with cardiovascular disease increased by 34.3%. As of 2016, 4 million Indians die annually due to NCDs prematurely occurring in the age range 30–70 years [23].

Medical costs incurred by workers in Qatar are significantly lower, even for those on minimum wage, not exceeding 2 days of their income, if requiring treatment for all NCDs (diabetes, hypertension and dyslipidaemia), compared to costs in their countries of origin. The costs of NCD treatments place a considerable burden on household income. Studies from India showed that high out-of-pocket expenditure for healthcare related to NCDs is significant in contributing to poverty [21,22]. An estimated 1.4 to 2 million Indians experienced catastrophic spending in 2004, and 600,000 to 800,000 people were impoverished by the costs of caring for cardiovascular disease and cancer [23]. The chronic nature of NCDs, and the projected increase in their prevalence, means that this economic impact is likely to grow cumulatively in the future. A review of medicine prices in two multi-country studies showed that in the public sector, it cost on average from two to eight days’ wages to purchase one month’s supply of at least one cardiovascular medicine [24], and one days’ wage to purchase one month’s supply of at least one anti-diabetic medicine [25]. One month of combination treatment for coronary heart disease costs 18.4 days’ wages in Malawi, 6.1 days’ wages in Nepal, 5.4 in Pakistan and 5.1 in Brazil. In India, paying for diabetes care can cost low-income households about one-third of their incomes [25]. The average annual direct cost of diabetes care in India could range from USD 484–535 per person, including outpatient visits, consultation, drugs, and admission due to complications [25]. The direct costs related to diabetes varies between uneducated and educated participants, from USD 9.2 to USD 64.8, suggesting a disparity in access to medical care.

The prevalence was 25.9%, 21.0%, 8.3%, and 28.4% for overweight and obesity, hypertension, diabetes, and hypercholesterolaemia, respectively, in a WHO-led survey of Bangladesh’s national population [26]. Mean annual number of visits to a diabetic outpatient clinic was 3.9 per year, and annual direct costs could range from USD 71 to USD 542, depending on complications [27]. 

The current study is based on data from patients’ electronic health records (EHRs) that were collected during the routine delivery of health care for the migrant workers registered at specific clinics. EHR data have been used to support observational studies, either as stand-alone data, as we have performed here, or following linkage to primary re-search data or, more recently, for recruitment to clinical trials [28]. These results represent clinical data, collected from health records designed for the delivery of primary healthcare, and specifically for the management of the health of workers across a spectrum of different diseases. It is increasingly recognized that EHRs facilitate primary healthcare screenings of patients by age, gender, and diagnosis, using criteria such as ICD-10, as well as being useful for the exclusion of ineligible patients in clinical trials [29]. Using EHRs as the source for demographic information, co-morbidities, and concomitant medications has several advantages over separately recording these data into electronic case reports, as transcription errors are reduced since EHR data are entered by providers directly involved in a patient’s care, as opposed to secondary entry by study personnel. Moreover, diagnosis by ICD-10 is a well-recognized and globally accepted criteria [20]. Therefore, we consider the measures used and the results both valid and reliable.

Our findings showed that migrant workers in Qatar have access to better and more affordable healthcare services, compared to their countries of origin. It is also apparent that migrant workers were predisposed to certain risk factors associated with living in Qatar, including, obesity, a sedentary lifestyle due to walking less and greater use of private and public transport, and exposure to heat stress related to the type and nature of their work [30]. Other unhealthy behaviors common among migrant workers, such as smoking, binge drinking, increased screen time, and unhealthy food choices are also likely, due to living without family [30]. Further research is needed to clearly determine whether living in Qatar contributed to the development of certain NCDs, or whether pre-existing conditions were exacerbated by factors encountered in the country. 

### Study Limitations

Although this study included a large sample of participants, it has some inherent limitations, in that it is retrospective and included only males. Socioeconomic class, nature of employment, and the ethnicities of female workers in Qatar have a different distribution to that of males, and therefore are better analyzed separately. The current project included only clinic visits with valid ICD-10 records and dates of first entry to Qatar to be able to calculate duration of residence. The cohort (n = 58,270) of male patients provided a representative sample of migrant workers across the country belonging to the blue-collar class. These results could guide future studies where prospective and longitudinal follow-up data would contribute to better understand the relationship between duration of residence and disease. The patient visits in this study were mostly confined to workers in proximity of the industrial area zone where the clinic was located; therefore, our study population was less likely to represent the class of workers with higher incomes or higher education statuses, who generally seek health counselling in the private sector. 

The sample size of the cohort with patients who had resided in Qatar for less than 6 months (n = 663) was another limitation. This group represents those who arrived with NCDs from their countries of origin, as opposed to those who developed it while in Qatar. As this is the group that all others are benchmarked against, increasing this pool would add greater strength to our findings. Screening programmes that include NCDs at entry into the country will help to increase this cohort and validate our findings. As the workers across countries of the GCC, including Saudi Arabia and UAE, are made up of predominantly South Asians from the Indian sub-continent, these findings can be generalized. 

Although we have shown the average cost of treatment ranging from 0.07 to 1.67 of days’ income, we did not have information on whether these healthcare costs were met by the employees of their respective employers. In addition, on 30 August 2020, the Ministry of Administrative Development, Labour and Social Affairs announced a new, enforceable minimum wage for workers (QAR 1800), which came into effect in February 2021 [31]. However, we believe that this is unlikely to influence the results of the study related to duration of residence, but the cost of treatment in the future will be presumably lower relative to income than what is reported in this study. Since only data from outpatient clinics were available for this study, emergency visits were not reported. Future research in these areas is warranted along with more detailed laboratory analyses.

## 5. Conclusions

Male, blue-collar workers in Qatar are predominantly South Asian, from lower socioeconomic classes, and typically have early onset NCDs, specifically diabetes and hypertension. Residence in Qatar gave them better access and affordable healthcare for effective management of these conditions. 

## Figures and Tables

**Table 1:** Characteristics of the study population.

Variables		Total No. of Patients (58,342)	
		n (%)	%
Age of the participant *			
	≤25	5318	9.1%
	25–35	21,037	36.1%
	35–45	18,806	32.3%
	45–55	10,243	17.6%
	55–65	2846	4.9%
Nationality			
	India	17,201	29.5%
	Nepal	16,687	28.6%
	Bangladesh	12,784	21.9%
	Sri Lanka	4082	7.0%
	Egypt	1968	3.4%
	Philippines	1743	3.0%
	Pakistan	1438	2.5%
	Kenya	530	0.9%
	Ghana	356	0.6%
	Others	1553	2.7%
Duration of residence in Qatar			
	<6 months (m)	663	1.1%
	6 m to ≤1 year	2308	4.0%
	1.1 to ≤2 years	9472	16.2%
	2.1 to ≤5 years	22,283	38.2%
	5.1 to ≤6 years	3907	6.7%
	>6 years	19,709	33.8%

* There were 92 cases in whom the variable “age” was missing. Percentages shown have taken this into account.

**Table 2:** Description of the population by diagnosis, duration of residence, age, number of visits and associated medical costs.

ICD Chapter	Total Patients (N = 58,342) n (%) *	Total Visits N = 119,581 n (%) *	Age of the Participants (Years)	Duration of Residence (Years)	Visits per Patient Mean ± SD Median [IQR]	Cost per Visit (QAR) Median [IQR]
E00–E89: Endocrine, nutritional and metabolic diseases	10,712 (18.4)	29,854 (25.0)	45.4 ± 8.4	9.6 ± 8.0	2.8 ± 1.3, 3 [2,4]	24 [11,46]
H00–H59: Diseases of the eye and adnexa	3552 (6.1)	4065 (3.4)	37.3 ± 9.7	6.3 ± 6.0	1.1 ± 0.5, 1 [1,1]	2 [1,4]
H60–H95: Diseases of the ear and mastoid process	1575 (2.7)	1806 (1.5)	35.4 ± 9.2	5.4 ± 5.0	1.1 ± 0.4, 1 [1,1]	2 [1,3]
I00–I99: Diseases of the circulatory system	7644 (13.1)	20,922 (17.5)	46.5 ± 8.4	10.0 ± 8.3	2.7 ± 1.3, 3 [2,4]	29 [12,48]
J00–J99: Diseases of the respiratory system	21,870 (37.5)	30,486 (25.5)	35.5 ± 9.3	5.3 ± 5.2	1.4 ± 0.8, 1 [1,2]	1 [1,3]
K00–K95: Diseases of the digestive system	13,897 (23.8)	19,347 (16.2)	36.2 ± 9.2	5.8 ± 5.5	1.4 ± 0.8, 1 [1,2]	2 [1,3]
L00–L99: Diseases of the skin and subcutaneous tissue	7591 (13.0)	10,015 (8.4)	35.7 ± 9.5	5.6 ± 5.5	1.3 ± 0.7, 1 [1,1]	2 [1,4]
M00–M99: Diseases of the musculoskeletal system and connective tissue	16,996 (29.1)	23,482 (19.6)	37.2 ± 9.3	5.9 ± 5.7	1.4 ± 0.8, 1 [1,2]	1 [1,2]
N00–N99: Diseases of the genitourinary system	3590 (6.2)	5259 (4.4)	35.9 ± 9.4	5.8 ± 5.5	1.5 ± 0.8, 1 [1,2]	2 [1,7]
S00–T88: Injury, poisoning and certain other consequences of external causes	7033 (12.1)	7806 (6.5)	34.4 ± 8.9	5.2 ± 4.8	1.1 ± 0.4, 1 [1,1]	1 [1,1]

* Percentages do not add to 100, because a patient might have more than one diagnosis. IQR: Inter Quartile Range. 1 QAR = 0.27 US$.

**Table 3:** Proportion of visits by ICD main and sub-group diagnosis related to duration of residence.

ICD Chapters	<6 Months	6–≤12 Months	1–≤2Years	2–≤5 Years	5–≤6 Years	>6 Years
	n (%)	n (%)	n (%)	n (%)	n (%)	n (%)
	N = 663	N = 2308	N = 9472	N = 22,283	N = 3907	N = 19,709
**E00–E89: Endocrine, nutritional and metabolic diseases**	148 (22.3) ^bcde^	222 (9.6)	739 (7.8)	2687 (12.1) ^bc^	623 (15.9) ^bcd^	6293 (31.9) ^abcde^
- E08–E13: Diabetes mellitus	110 (16.6) ^bcde^	160 (6.9)	512 (5.4)	1792 (8.0) ^c^	418 (10.7) ^bcd^	4361 (22.1) ^abcde^
- E65–E68: Overweight, obesity and other hyperalimentation	19 (2.9) ^c^	34 (1.5)	112 (1.2)	410 (1.8) ^c^	96 (2.5) ^e^	1101 (5.6) ^abcde^
- E70–E88: Metabolic disorders	109 (16.4) ^bcde^	152 (6.6)	515 (5.4)	1983 (8.9) ^bc^	453 (11.6) ^bcd^	4904 (24.9) ^abcde^
- E00–07;15–16;20–35;50–64;89 Others	8 (1.2)	10 (0.4)	33 (0.3)	97 (0.4)	21 (0.5)	153 (0.8)
**H00–H59: Diseases of the eye and adnexa**	35 (5.3)	164 (7.1) ^c^	515 (5.4)	1325 (5.9)	235 (6.0)	1278 (6.5) ^c^
**H60–H95: Diseases of the ear and mastoid process**	16 (2.4)	84 (3.6) ^f^	278 (2.9)	620 (2.8)	112 (2.9) ^f^	465 (2.4)
**I00–I99: Diseases of the circulatory system**	81 (12.2) ^bcd^	146 (6.3)	528 (5.6)	1793 (8.0) ^c^	453 (11.6) ^bcd^	4643 (23.6) ^abcde^
- I10–I16: Hypertensive diseases	76 (11.5) ^bcd^	128 (5.5)	455 (4.8)	1615 (7.2) ^bc^	418 (10.7) ^bcd^	4431 (22.5) ^abcde^
- I20–I25: Ischemic heart diseases	5 (0.8)	9 (0.4)	29 (0.3)	106 (0.5)	16 (0.4)	257 (1.3) ^bcde^
- I00–02;05–09;26–28;30–52;60–89;95–99 Others	3 (0.5)	16 (0.7)	65 (0.7)	150 (0.7)	32 (0.8)	184 (0.9) ^d^
**J00–J99: Diseases of the respiratory system**	254 (38.3) ^ef^	1082 (46.9) ^adef^	4204 (44.4) ^adef^	8607 (38.6) ^ef^	1384 (35.4)	6339 (32.2) ^e^
**K00–K95: Diseases of the digestive system**	170 (25.6)	586 (25.4) ^f^	2361 (24.9) ^f^	5382 (24.2) ^f^	941 (24.1)	4457 (22.6)
**L00–L99: Diseases of the skin and subcutaneous tissue**	85 (12.8)	336 (14.6) ^f^	1315 (13.9) ^f^	3005 (13.5) ^f^	494 (12.6)	2356 (12.0)
**M00–M99: Diseases of the musculoskeletal system and connective tissue**	187 (28.2)	765 (33.1) ^cdef^	2713 (28.6) ^e^	6635 (29.8) ^ef^	1102 (28.2)	5594 (28.4) ^e^
**N00–N99: Diseases of the genitourinary system**	56 (8.4)	170 (7.4)	598 (6.3)	1370 (6.1)	244 (6.2)	1152 (5.8)
**S00–T88: Injury, poisoning and certain other consequences of external causes**	76 (11.5)	321 (13.9) ^f^	1280 (13.5) ^f^	2943 (13.2) ^f^	501 (12.8) ^f^	1912 (9.7)

a: significantly higher than <6 months; b: significantly higher than 6–≤12 months; c: significantly higher than 1–≤2 years; d: significantly higher than 2–≤5 years; e: significantly higher than 5–≤6 years; f: significantly higher than >6years.

**Table 4:** The estimated cost per visit of treatment for NCDs.

	Total Cost per Visit/ Every 2 Months	
	Paid by Ministry of Public Health	Paid by Patient
IM Specialist consultation	84.19	0
Nurse	29.00	0
Laboratory tests	49.40	0
Medication for diabetes	118.00	23.60
Medication for hypertension	66.00	13.20
Medication for lipids	7.70	1.54
Miscellaneous consumable items	8.70	0
ECG	1.80	0
Total Direct Cost	364.79	-
Total Indirect Cost	159.25	-
Overall Total Cost	524.04	38.34

## Data Availability

The dataset used/analyzed for this study is with the corresponding author and can be made available with limited capacity upon request.

## Supplementary Materials

The following are available online at https://www.mdpi.com/article/10.3390/ijerph19042266/s1, Figure S1: Population pyramid of Qatar by sex. Table S1: Proportion of visits by ICD diagnosis according to duration of residence in Qatar among blue-collar workers during study the period January 2017 to May 2018.
