# Supplementary material for: A Retrospective Study of Non-Communicable Diseases amongst Blue-Collar Migrant Workers in Qatar
Source: Int J Environ Res Public Health. 2022 Feb 17;19(4):2266. doi: 10.3390/ijerph19042266 (PMC8872334; doi:10.3390/ijerph19042266)
Supplement: Supplementary file 1 [file ijerph-19-02266-s001.zip › Supplementary Table S1.pdf]

Supplementary Table S1. Proportion of visits by ICD diagnosis according to duration of residence in Qatar among blue collar workers during study the period Jan 2017 to May 2018.

	<6 months	6-≤12 months	1 - ≤2 years	2 - ≤5 years	5 - ≤6 years	> 6 years
	n(%)	n(%)	n(%)	n(%)	n(%)	n(%)
**ICD Sub chapters**	**A**	**B**	**C**	**D**	**E**	**F**
**E00-E89: Endocrine, nutritional and metabolic diseases**	**148(22.3)**	**222(9.6)**	**739(7.8)**	**2687(12.1)^{c}**	**623(15.9)^{bcd}**	**6293(31.9)^{abcde}**
E08-E13: Diabetes mellitus	110(16.6) ^{bcde}	160(6.9)	512(5.4)	1792(8.0)^{c}	418(10.7)^{bcd}	4361(22.1)^{abcde}
E65-E68: Overweight, obesity and other hyperalimentation	19(2.9)^{c}	34(1.5)	112(1.2)	410(1.8)^{c}	96(2.5)^{e}	1101(5.6)^{abcde}
E70-E88: Metabolic disorders	109(16.4)^{bcde}	152(6.6)	515(5.4)	1983(8.9)^{bc}	453(11.6)^{bcd}	4904(24.9)^{abcde}
E00-07;15-16;20-35;50-64;89 Others	8(1.2) ^{c}	10(0.4)	33(0.3)	97(0.4)	21(0.5)	153(0.8) ^{cd}
**H00-H59: Diseases of the eye and adnexa**	**35(5.3)**	**164(7.1)**	**515(5.4)**	**1325(5.9)**	**235(6.0)**	**1278(6.5)**
H00-H05: Disorders of eyelid, lacrimal system and orbit	20(3.0)	83(3.6)	288(3.0)	731(3.3)	134(3.4)	593(3.0)
H10-H11: Disorders of conjunctiva	1(0.2)	19(0.8)	59(0.6)	181(0.8)	36(0.9)	155(0.8)
H15-H22: Disorders of sclera, cornea, iris and ciliary body	3(0.5)	14(0.6)	48(0.5)	90(0.4)	24(0.6)	107(0.5)
H49-H52: Disorders of ocular muscles, binocular movement, accommodation and refraction	8(1.2)	26(1.1)	65(0.7)	203(0.9)	32(0.8)	333(1.7)^{cde}
H59-H59: Intraoperative and postprocedural complications and disorders of eye and adnexa, not elsewhere classified	4(0.6)	28(1.2)	78(0.8)	195(0.9)	27(0.7)	170(0.9)
**H60-H95: Diseases of the ear and mastoid process**	**16(2.4)**	**84(3.6)**	**278(2.9)**	**620(2.8)**	**112(2.9)**	**465(2.4)**
H60-H62: Diseases of external ear	3(0.5)	27(1.2)^{f}	81(0.9)	163(0.7)	30(0.8)	115(0.6)
H65-H75: Diseases of middle ear and mastoid	8(1.2)	38(1.6)^{f}	105(1.1)	243(1.1)^{f}	45(1.2)	156(0.8)
H80-H83: Diseases of inner ear	3(0.5)	15(0.6)	56(0.6)	152(0.7)	32(0.8)	160(0.8)
H90-H94: Other disorders of ear	3(0.5)	14(0.6)	61(0.6)^{f}	97(0.4)	13(0.3)	63(0.3)
H25-28;30-36;40-44;46-47;56-57 Others	1(0.2)	11(0.5)	26(0.3)	88(0.4)	9(0.2)	107(0.5)^{c}
**I00-I99: Diseases of the circulatory system**	**81(12.2)**	**146(6.3)**	**528(5.6)**	**1793(8.0)**	**453(11.6)**	**4643(23.6)**
I10-I16: Hypertensive diseases	76(11.5)^{bcd}	128(5.5)	455(4.8)	1615(7.2)^{bc}	418(10.7)^{bcd}	4431(22.5)^{abcde}
I20-I25: Ischemic heart diseases	5(0.8)	9(0.4)	29(0.3)	106(0.5)	16(0.4)	257(1.3)^{bcde}
I00-02;05-09;26-28;30-52;60-89;95-99 Others	3(0.5)	16(0.7)	65(0.7)	150(0.7)	32(0.8)	184(0.9)^{d}
**J00-J99: Diseases of the respiratory system**	**254(38.3)**	**1082(46.9)**	**4204(44.4)**	**8607(38.6)**	**1384(35.4)**	**6339(32.2)**

J00-J06: Acute upper respiratory infections	215(32.4)[f]	937(40.6)[adef]	3552(37.5)[def]	7067(31.7)[ef]	1098(28.1)[f]	4815(24.4)
J09-J18: Influenza and pneumonia	4(0.6)	29(1.3)[f]	131(1.4)[def]	198(0.9)	28(0.7)	135(0.7)
J20-J22: Other acute lower respiratory infections	32(4.8)	148(6.4)[f]	521(5.5)	1181(5.3)	209(5.3)	982(5.0)
J30-J39: Other diseases of upper respiratory tract	24(3.6)	74(3.2)	323(3.4)	828(3.7)[f]	110(2.8)	615(3.1)
J40-J47: Chronic lower respiratory diseases	18(2.7)	38(1.6)	180(1.9)	473(2.1)	103(2.6)	774(3.9)[bcde]
J60-70;80-86;90-94;96-99 Others	1(0.2)	3(0.1)	7(0.1)	22(0.1)	4(0.1)	14(0.1)
**K00-K95: Diseases of the digestive system**	**170(25.6)**	**586(25.4)**	**2361(24.9)**	**5382(24.2)**	**941(24.1)**	**4457(22.6)**
K00-K14: Diseases of oral cavity and salivary glands	68(10.3)	214(9.3)	994(10.5)	2307(10.4)	399(10.2)	1948(9.9)
K20-K31: Diseases of esophagus, stomach and duodenum	76(11.5)	284(12.3)[def]	975(10.3)[f]	2222(10.0)[f]	346(8.9)	1648(8.4)
K55-K64: Other diseases of intestines	2(0.3)	5(0.2)	29(0.3)[f]	47(0.2)	13(0.3)	26(0.1)
K35-38;40-46;50-52;70-77;80-87;90-95 Others	38(5.7)	185(8.0)[d]	612(6.5)	1395(6.3)	258(6.6)	1269(6.4)
**L00-L99: Diseases of the skin and subcutaneous tissue**	**85(12.8)**	**336(14.6)**	**1315(13.9)**	**3005(13.5)**	**494(12.6)**	**2356(12.0)**
L00-L08: Infections of the skin and subcutaneous tissue	24(3.6)	68(2.9)	264(2.8)	659(3.0)[f]	111(2.8)	471(2.4)
L20-L30: Dermatitis and eczema	36(5.4)	172(7.5)	602(6.4)	1424(6.4)	250(6.4)	1226(6.2)
L40-L45: Papulosquamous disorders	4(0.6)	10(0.4)	43(0.5)	148(0.7)	19(0.5)	103(0.5)
L49-L54: Urticaria and erythema	15(2.3)	47(2.0)	161(1.7)	343(1.5)	54(1.4)	301(1.5)
L60-L75: Disorders of skin appendages	8(1.2)	42(1.8)[f]	189(2.0)[ef]	368(1.7)[f]	49(1.3)	209(1.1)
L80-L99: Other disorders of the skin and subcutaneous tissue	9(1.4)	32(1.4)	180(1.9)[f]	351(1.6)	52(1.3)	276(1.4)
L10-14;55-59	0(0.0)	1(0.0)	11(0.1)	11(0.0)	2(0.1)	18(0.1)
**M00-M99: Diseases of the musculoskeletal system and connective tissue**	**187(28.2)**	**765(33.1)**	**2713(28.6)**	**6635(29.8)**	**1102(28.2)**	**5594(28.4)**
M05-M14: Inflammatory polyarthropathies	2(0.3)	18(0.8)	55(0.6)	107(0.5)	26(0.7)	174(0.9)[d]
M15-M19: Osteoarthritis	2(0.3)	16(0.7)	28(0.3)	92(0.4)	20(0.5)	289(1.5)[bcde]
M20-M25: Other joint disorders	36(5.4)	135(5.8)	438(4.6)	1116(5.0)	198(5.1)	1207(6.1)[cd]
M50-M54: Other dorsopathies	55(8.3)	268(11.6)[f]	908(9.6)	2254(10.1)[f]	367(9.4)	1716(8.7)
M60-M63: Disorders of muscles	9(1.4)	54(2.3)[f]	188(2.0)[f]	482(2.2)[f]	71(1.8)	296(1.5)
M70-M79: Other soft tissue disorders	113(17.0)	431(18.7)[cdef]	1488(15.7)	3565(16.0)[f]	584(14.9)	2843(14.4)
M00-02;26-27;30-36;40-43;45-49;65-67;80-94;96;99 Others	6(0.9)	29(1.3)	76(0.8)	186(0.8)	37(0.9)	176(0.9)
**N00-N99: Diseases of the genitourinary system**	**56(8.4)**	**170(7.4)**	**598(6.3)**	**1370(6.1)**	**244(6.2)**	**1152(5.8)**
N20-N23: Urolithiasis	36(5.4)	115(5.0)	412(4.3)	935(4.2)	163(4.2)	753(3.8)

N30-N39: Other diseases of the urinary system	13(2.0)	35(1.5)	138(1.5)	296(1.3)	57(1.5)	268(1.4)
N40-N53: Diseases of male genital organs	7(1.1)	30(1.3)	94(1.0)	206(0.9)	39(1.0)	189(1.0)
N00-08; 10-19; 25-29; 60-65 Others	4(0.6)	7(0.3)	15(0.2)	49(0.2)	18(0.5)[c]	93(0.5)[cd]
**S00-T88: Injury, poisoning and certain other consequences of external causes**	**76(11.5)**	**321(13.9)**	**1280(13.5)**	**2943(13.2)**	**501(12.8)**	**1912(9.7)**
S00-S09: Injuries to the head	5(0.8)	32(1.4)	138(1.5)	311(1.4)	60(1.5)	226(1.1)
S10-S19: Injuries to the neck	2(0.3)	3(0.1)	9(0.1)	32(0.1)	14(0.4)[cdf]	18(0.1)
S20-S29: Injuries to the thorax	2(0.3)	12(0.5)	40(0.4)	112(0.5)	15(0.4)	78(0.4)
S30-S39: Injuries to the abdomen, lower back, lumbar spine, pelvis and external genitals	4(0.6)	21(0.9)	76(0.8)	159(0.7)	27(0.7)	110(0.6)
S40-S49: Injuries to the shoulder and upper arm	4(0.6)	5(0.2)	30(0.3)	84(0.4)	11(0.3)	63(0.3)
S50-S59: Injuries to the elbow and forearm	7(1.1)	19(0.8)[f]	64(0.7)[f]	102(0.5)[f]	22(0.6)	66(0.3)
S60-S69: Injuries to the wrist, hand and fingers	24(3.6)	113(4.9)[f]	448(4.7)[f]	1018(4.6)	164(4.2)[f]	596(3.0)
S70-S79: Injuries to the hip and thigh	0(0.0)	3(0.1)	13(0.1)	32(0.1)	6(0.2)	17(0.1)
S80-S89: Injuries to the knee and lower leg	3(0.5)	32(1.4)	108(1.1)	235(1.1)	40(1.0)	165(0.8)
S90-S99: Injuries to the ankle and foot	18(2.7)	66(2.9)[f]	239(2.5)[f]	566(2.5)[f]	93(2.4)	367(1.9)
T15-T19: Effects of foreign body entering through natural orifice	9(1.4)	34(1.5)	156(1.6)f	333(1.5)f	51(1.3)	199(1.0)
T20-T25: Burns and corrosions of external body surface, specified by site	1(0.2)	3(0.1)	25(0.3)	70(0.3)	15(0.4)	46(0.2)
T07;14;26-28;30-32;36-50;51-78;80-88 Others	4(0.6)	6(0.3)	21(0.2)	62(0.3)	15(0.4)	62(0.3)
