# Supplementary figures and images for: A Retrospective Study of Non-Communicable Diseases amongst Blue-Collar Migrant Workers in Qatar
Source: Int J Environ Res Public Health. 2022 Feb 17;19(4):2266. doi: 10.3390/ijerph19042266 (PMC8872334; doi:10.3390/ijerph19042266)

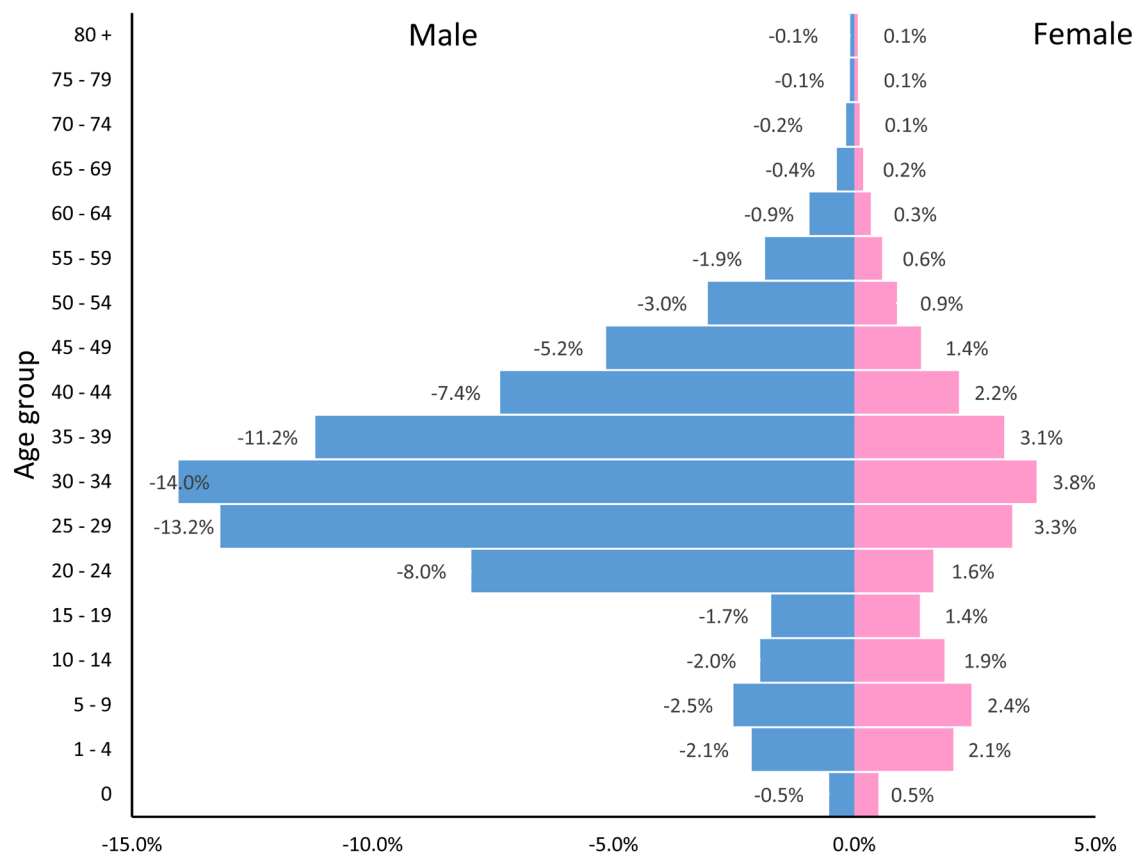

**Supplementary Figure S1. Population pyramid of Qatar by sex**

Data based on census 2018, N=2,760,170

Supplement: Supplementary file 1 [file ijerph-19-02266-s001.zip › Supplementary Figure S1.pdf]
